# BRCA somatic and germline mutation detection in paraffin embedded ovarian cancers by next-generation sequencing

**DOI:** 10.18632/oncotarget.6834

**Published:** 2016-01-07

**Authors:** Andrea Mafficini, Michele Simbolo, Alice Parisi, Borislav Rusev, Claudio Luchini, Ivana Cataldo, Elena Piazzola, Nicola Sperandio, Giona Turri, Massimo Franchi, Giampaolo Tortora, Chiara Bovo, Rita T. Lawlor, Aldo Scarpa

**Affiliations:** ^1^ ARC-Net Research Centre, University and Hospital Trust of Verona, Verona, Italy; ^2^ Department of Pathology & Diagnostics, University and Hospital Trust of Verona, Verona, Italy; ^3^ Department of Gynecology, University and Hospital Trust of Verona, Verona, Italy; ^4^ Comprehensive Cancer Centre, University and Hospital Trust of Verona, Verona, Italy; ^5^ Board of Directors, University and Hospital Trust of Verona, Verona, Italy

**Keywords:** BRCA1-BRCA2, ovarian carcinoma, next generation sequencing, PARP inhibitor, olaparib

## Abstract

*BRCA* mutated ovarian cancers respond better to platinum-based therapy and to the recently approved PARP-inhibitors. There is the need for efficient and timely methods to detect both somatic and germline mutations using formalin-fixed paraffin-embedded (FFPE) tissues and commercially available technology. We used a commercial kit exploring all exons and 50bp exon-intron junctions of *BRCA1* and *BRCA2* genes, and semiconductor next-generation sequencing (NGS) on DNA from 47 FFPE samples of high-grade serous ovarian cancers. Pathogenic mutations were found in 13/47 (28%) cancers: eight in *BRCA1* and five in *BRCA2*. All *BRCA1* and two *BRCA2* mutations were germline; three *BRCA2* mutations were somatic. All mutations were confirmed by Sanger sequencing. To evaluate the performance of the NGS panel, we assessed its capability to detect the 6,953 variants described for *BRCA1* and *BRCA2* in ClinVar and COSMIC databases using callability analysis. 6,059 (87.1%) variants were identified automatically by the software; 829 (12.0%) required visual verification. The remaining 65 (0.9%) variants were uncallable, and would require 15 Sanger reactions to be resolved. Thus, the sensitivity of the NGS-panel was 99.1%. In conclusion, NGS performed with a commercial kit is highly efficient for detection of germline and somatic mutations in *BRCA* genes using routine FFPE tissue.

## INTRODUCTION

Ovarian cancer is the most deadly tumour of the female reproductive system with 238,700 new cases and 151,900 deaths worldwide [1-3], of which 65,500 new cases and 42,700 deaths in Europe in 2012 [4]. In Italy, ovarian cancer accounts for 30% of all tumours of the female genital apparatus, and in 2013 there were 4,800 new cases and 37,829 prevalent cases [5].

*BRCA1* and *BRCA2* are among the most frequently mutated genes in high-grade ovarian serous carcinoma, which is responsible for the vast majority of ovarian cancer deaths [6, 7]. *BRCA1* and *BRCA2* genes are key partners of the homologous recombination (HR) DNA repair system, together with *ATM*, *BARD1*, *NBN* and other genes [6]. Indeed, germline and somatic mutations in HR genes occur in about 30% of patients with ovarian carcinoma, of which up to 75% are in *BRCA1* and *BRCA2* genes [6, 8].

Patients carrying a germline or somatic *BRCA1/BRCA2* mutation have been associated with a better prognosis and a better response to platinum-based therapy [8-11]. A particular class of drugs, poly(ADP-ribose) polymerase-inhibitors (PARPi), has been shown to be effective for targeted treatment of cancers harbouring *BRCA1* or *BRCA2* mutations [12-18]. The PARP-1 protein is critical to the repair of single-strand DNA breaks. In cells with defective HR, such as the *BRCA* mutations carriers, PARP-1 inhibition is synthetic lethal and results in cell cycle arrest and subsequent apoptosis [15]. On December 2014 the European Medicines Agency (EMA) and the U.S.A. Food and Drug Administration (FDA) approved the PARPi olaparib [13-15] for treatment of *BRCA1/BRCA2* mutated ovarian cancer.

Investigating *BRCA* mutational status in ovarian cancer patients has thus a key role, not only for the identification of familial cancer predisposition but also to address therapeutic choices. Germline testing of *BRCA* is widespread in medical genetics laboratories, but this approach excludes patients with somatic *BRCA* mutations, i.e. only present in cancer cells, from the opportunity to avail of PARPi therapies. Testing *BRCA* on formalin-fixed paraffin-embedded (FFPE) samples would permit the simultaneous assessment of both somatic and germline mutations using an easily accessible material that is routinely available in any pathology laboratory worldwide.

High throughput next-generation sequencing (NGS) technologies permit fast multiplex testing on small quantities of DNA with budding applications in cancer diagnostics as they improve both the capacity and the cost-effectiveness of mutational analysis compared with Sanger [19-23]. To assess the feasibility of using NGS in routine diagnostic activity for *BRCA* analysis, we have investigated *BRCA1* and *BRCA2* mutations in 47 high-grade serous tumours of the ovary, using a commercially available kit and semiconductor NGS on FFPE tissue samples.

## RESULTS

**Figure 1:**
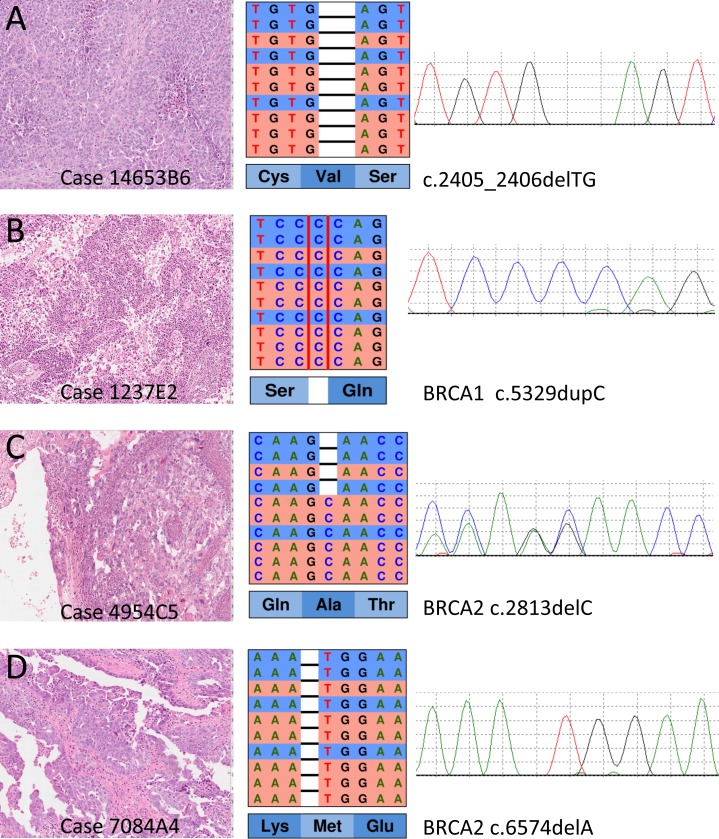
Representative examples of mutations detected at next generation sequencing with the HR1 kit and validated by Sanger sequencing On the left, the histological section of the primary ovarian cancer (Hematoxylin and eosin stain) from which DNA has been prepared after microdisection of the most cellular areas. In the middle, the representation of the results of next-generation sequencing where the reads (red for forward and blue for reverse) are aligned to the reference genome as provided by the Integrative Genomics Viewer (IGV v.2.3, Broad Institute) software. On the right, the representation of Sanger sequencing results for each cancer to validate the mutations (forward strand was used in **A**. **B**. and **D**.; reverse strand was used in **C**.). The *BRCA1* mutations in A, B and the *BRCA2* mutation in D are homozygous in cancer tissue as shown in both IGV and Sanger representations; these mutations were heterozygous in germline DNA of the respective patients. The *BRCA2* mutation in C was heterozygous in tumour tissue, and its somatic nature was determined by its absence in matching normal DNA (not shown).

The results of NGS targeted sequencing are reported in Table 1 and an example is shown Figure 1. DNA from all samples was successfully amplified in multiplex PCR and an adequate library for NGS was obtained. The mean read length was 112 base pairs and a mean coverage of 3,507x was achieved, with 99.6% target bases covered more than 100x.

**Table 1:** Pathogenic mutations in *BRCA1* and *BRCA2* detected by next-generation sequencing of 47 ovarian cancers

Case	*BRCA1*	*BRCA2*	Mutation type	Germline-somatic	dbSNP ID	ClinVar class
3506	c.5329dupC p.Gln1777ProfsTer74	-	Frameshift	Germline	rs397507247	Pathogenic
3513	c.676delT p.Cys226ValfsTer8	-	Frameshift	Germline	rs80357941	Pathogenic
3508	c.1687C>T p.Gln563Ter	-	Nonsense	Germline	rs80356898	Pathogenic
3521	c.2405_2406delTG p.Val802GlufsTer7	-	Frameshift	Germline	rs80357706	Pathogenic
3528	c.2405_2406delTG p.Val802GlufsTer7	-	Frameshift	Germline	rs80357706	Pathogenic
3489	c.3767_3768delCA p.Thr1256ArgfsTer10	-	Frameshift	Germline	rs730881440	Pathogenic
3505	c.5125_5127delGTT p.Val1709del	-	In-frame deletion	Germline	rs80358344	Pathogenic
3520	c.5309C>T p.Pro1770Leu	-	Missense	Germline	-	-*
3512	-	c.2813delC p.Ala938GlufsTer22	Frameshift	Somatic	-	-**
3523	-	c.6202dupA p.Ile2068AsnfsTer10	Frameshift	Germline	rs397507833	Pathogenic
3514	-	c.6574delA p.Met2192TrpfsTer14	Frameshift	Germline	-	-**
3501	-	c.7069_7070delCT p.Leu2357ValfsTer2	Frameshift	Somatic	rs80359636	Pathogenic
3516	-	c.8614G>T p.Glu2872Ter	Nonsense	Somatic	-	-**

* This variant is not recorded on CinVar, but a variant on the very same codon, ClinVar variant :c.5309C>G (p.Pro1770Arg, rs80357462) is recorded as Pathogenic.

** These variants are not recorded in either dbSNP or ClinVar, however they cause a premature stop codon, which is a feature of pathogenic mutations.

### Pathogenic variants

Pathogenic mutations in *BRCA* genes were found in 13 of the 47 (28%) ovarian cancers: eight were in *BRCA1* and five in *BRCA2* (Table 1).

Of the eight mutations in *BRCA1* gene, five were frame-shifts resulting in a premature stop codon, one was a nonsense mutation, one was an in-frame single codon deletion and one was a missense mutation. Frame-shift, nonsense and in-frame deletion mutations are recorded as pathogenic in the ClinVar database; the only missense mutation found (c.5309C>T; p.Pro1770Leu) has not yet been recorded, but falls in the same codon where a pathogenic missense mutation (c.5309C>G; p.Pro1770Arg) is recorded in ClinVar (Table 1). All of the *BRCA1* mutations were germline as assessed by the analysis of DNA from matched normal tissue.

Of the five *BRCA2* mutations, four were frame-shifts (three deletions and one insertion) resulting in a premature stop codon and one was a nonsense point mutation. Three of the *BRCA2* mutations were somatic and two were germline as assessed by the analysis of matched normal tissue DNA (Table 1). One of the somatic (c.7069_7070delCT) and one of the germline (c.6202dupA) mutations are recorded as pathogenic in the ClinVar database. The remaining two somatic and one germline *BRCA2* mutations have not yet been recorded but were considered pathogenic as they cause a premature stop codon, a feature of pathogenic mutations.

All the above variants were confirmed at Sanger sequencing (Figure 1), therefore our estimated specificity was 100%.

### Additional variants

In the *BRCA1* gene, an additional germline variant, c.3119G>A (p.Ser1040Asn), was found in two cases. This variant is recorded as benign/likely benign in the ClinVar database by eight submitters, as uncertain by one and as pathogenic by one; its frequency in the global population is 1% according to the 1000Genomes project database [24]. In the *BRCA2* gene, two germline single nucleotide polymorphisms (SNP) were also detected in 29 patients: 24 harboured the c.1114A>C (p.Asn372His) SNP, three the c.865A>C (p.Asn289His) SNP, and two patients had both. The first SNP, c.1114A>C (p.Asn372His), has been recorded as benign in the ClinVar database by four submitters and as pathogenic by one; its frequency in the global population is 25% according to the 1000Genomes project database. The second SNP, c.865A>C (p.Asn289His), has been recorded as benign by seven submitters; its frequency in the global population is 7%.

### Sensitivity of targeted NGS

To evaluate the performance of the NGS panel, we assessed its capability to detect the 6,953 variants described for *BRCA1* and *BRCA2*, comprising 6,106 germline variants in the ClinVar database and 1,071 somatic mutations in COSMIC database, of which 224 overlap (Table 2).

**Table 2:** Somatic and Germline BRCA mutation callability analysis of HR1 Next-Generation kit vs Sanger sequencing

GENE	Coding region (bp)	ClinVar – COSMIC Variants^*^	Total variants^*^	N. Sanger needed^$^	Callability ^°^ of HR1 Next-Generation Sequencing and Variant Caller software			
					AutomaticCalls	Called by IGV^**^	Uncallable^***^	Sensitivity (%)
BRCA1	5,659	2,522 - 398	2,841	55	2,520	320	1	99.9
BRCA2	10,262	3,584 - 673	4,112	77	3,539	509	64	98.4
Total	15,921	6,106 - 1,071	6,953	132	6,059	829	65	99.1

° Callability analyis using the Torrent Variant Caller.

* Germline variants listed in the ClinVar database (http://www.ncbi.nlm.nih.gov/clinvar/) and mutations in COSMIC database (http://cancer.sanger.ac.uk/cancergenome/projects/cosmic/). The number of total variants is less than the sum of variants from both databases (7177) because 224 variants overlap.

$ Estimate based on one reaction per 150 bp (one per exon if <150 bp) using DNA from formalin-fixed paraffin-embedded tissue

** Visual verification of sequences with Integrative Genomics Viewer (IGV v.2.3, Broad Institute) software.

*** Mutations within homopolymer stretches, artefact-prone regions of the genes, or not covered by the NGS panel. The number of Sanger to solve these 65 blind spots would be 1 for *BRCA1* and 14 for *BRCA2*.

Analysis of callable/uncallable/poorly mapped loci was performed to discriminate between variants which can be automatically detected by the software from those that are hindered by sequencing errors due to homopolymers or PCR amplification artefacts. The procedure is described in detail in the Methods section. This analysis showed that the HR1 NGS-kit obtained a clear sequence of the DNA regions harbouring 6059 (87.1%) of these variants that were automatically identified by the Variant Caller Plugin software (Torrent Suite Software v4.6; Life Technologies), while the regions harbouring the remaining 894 (12.9%) variants were challenging for automatic detection. Of these, 829 (12.0%) variants were automatically identified by the software but with low confidence due to their proximity to homopolymer stretches or artefact-prone regions and thus required confirmation/correction by visual inspection of the region using the Integrative Genomics Viewer (IGV) v2.3 (Broad Institute). In detail, the number of variants that required visual inspection to be confirmed was 320 for *BRCA1* and 509 for *BRCA2*. The remaining 65 (0.9%) variants were uncallable. Of these 65, 13 (0.2%) were single/double base insertion or deletions located within homopolymer stretches, 1 for *BRCA1* and 12 for *BRCA2*, and both software and visual inspection were insufficient to discern between an artefact and a true alteration. The remaining 52 (0.7%) variants reside in regions of *BRCA2* that are difficult to amplify by current NGS library approaches for FFPE tissue, and thus could not be amplified in HR1 panel. Uncallable variants would require 15 Sanger sequencing reactions to be resolved (Table 2). In conclusion, the sensitivity of *BRCA1* and *BRCA2* NGS sequencing was 99.1%.

## DISCUSSION

The assessment of *BRCA* mutational status in ovarian cancer patients plays a double role. The first is the identification of familial cancer predisposition; the second is to address therapeutic choices.

*BRCA1* and *BRCA2* germline mutations are known risk factors for ovarian cancer [2, 7, 25-33], and it has been reported that up to 44% of patients without a family history of ovarian cancer harbour a germline *BRCA1* or *BRCA2* mutation, and as such may act as first alert for descendants [10].

The better response to platinum and the recent approval of PARP-inhibitors for therapy of ovarian cancers harbouring mutations in *BRCA* genes calls for methods able to detect not only germline but also somatic mutations in these genes.

Molecular tests to analyse *BRCA* mutations are generally carried out on germline DNA from blood samples to identify hereditary mutations as part of risk assessment programs. However, in order to identify somatic mutations present in neoplastic cells to permit the patient to benefit from drugs such as olaparib, it is necessary to analyze DNA from the tumour tissue.

The analysis of BRCA on diagnostic tissue is complex due to: 1) the type of possible mutations in these genes; 2) the fact that these mutations may be found in any part of these genes which are very large and as such require the entire gene to be sequenced; and 3) the limited quantity and low quality of DNA available from routine diagnostic FFPE tissue is limited. It is therefore important to be able to sequence the entire *BRCA* genes using minimal amounts of DNA to identify pathogenic mutations relevant to patient treatment.

In this study, we tested a commercially available NGS sequencing panel on small quantities of DNA purified from FFPE tissue to evaluate the use of these technologies for diagnostic applications.

The analysis of 47 high grade serous ovarian cancers identified 13 (28%) pathogenic mutations, 8 *BRCA1* and 5 *BRCA2*. All *BRCA1* and two *BRCA2* mutations were germline while three *BRCA2* mutations were somatic. *BRCA1* and *BRCA2* mutations were mutually exclusive. All 13 *BRCA* variants were confirmed by Sanger sequencing, demonstrating an estimated specificity of 100%.

To evaluate sensitivity of the NGS panel, we assessed its capability to detect all 6,953 germline or somatic variants described for *BRCA1* and *BRCA2* in the ClinVar and COSMIC databases using the analysis of callable loci (details in Methods section). A total of 6,059 (87.1%) of these variants were automatically identified by the Torrent Variant Caller software. A further 829 (12.0%) were imprecisely identified by the software and required confirmation/correction by visual inspection of the region. This was due to the mutations being either inside or in close proximity of homopolymer stretches or PCR amplification artefacts. The remaining 65 (0.9%) variants were uncallable resulting in the sensitivity of the HR1 NGS-panel at 99.1%, as it would miss 65/6,953 variants. These could be resolved, however, with 15 Sanger sequencing reactions.

In conclusion, our study shows that next-generation sequencing performed with a commercial kit (HR1, 4Bases) is highly efficient for detection of germline and somatic mutations in *BRCA1* and *BRCA2* genes using DNA from routinely available FFPE tissue.

## MATERIALS AND METHODS

### Cases

The study series comprised 47 samples of high grade serous ovarian carcinomas, diagnosed according to WHO classification criteria [2], from patients who underwent surgical resection between 2010 and 2015 (mean age 61±12 years, median 62 years) at the Department of Obstetrics and Gynaecology of the University Hospital Trust of Verona. Five patients were diagnosed at stage IV, 35 at stage IIIC, 4 at stage I and 3 at stage IC, according to FIGO staging system [34].

### Ethics

The samples were acquired from the Integrated University Hospital Trust of Verona Pathology archives under the amended Program 1885, that permits the acquisition of FFPE samples by the ARC-Net (Applied Research on Cancer) biobank of the University of Verona and the Hospital Trust of Verona following the re-consent of patients or the anonymization of samples under protocol 52438 Prog. 1885 approved 23/11/2010. The amendment also addresses the regulatory issue of data protection and disclosure in genomic studies. Subsequent approval for this study was presented and approved under protocol 44541 on 29/09/2015.

### DNA extraction and qualification

FFPE samples of ovarian cancers were enriched for neoplastic cellularity to a minimum of 70% by manual microdissection of 10 consecutive 4-μm sections. DNA was purified using the QIAamp FFPE Tissue Kit (Qiagen) and qualified as previously reported [35, 36]. Briefly, DNA was quantified using Qubit (LifeTechnologies) platforms, and its quality was further evaluated by NanoDrop (Life Technologies) and PCR analysis using the BIOMED 2 PCR multiplex protocol [36].

### Deep sequencing of multiplex PCR amplicons

The HR1 kit (4bases SA, Switzerland) was used. This kit explores all exons of *BRCA1* (n=24; NM_007300.3) and *BRCA2* (n=27; NM_000059.3) and 50 bp exon-intron junctions. The kit is composed of three multiplex PCR primer pools, and uses 30 nanograms of DNA, ten per primer pool, for multiplex PCR amplification, followed by ligation of a specific barcode-sequence to each sample for identification The quality of the obtained libraries was evaluated by the Agilent 2100 Bioanalyzer on-chip electrophoresis (Agilent Technologies) as previously described [35]. Emulsion PCR for clonal amplification of libraries was performed with the Ion OneTouch OT2 System and the Hi-Q OT2 200 Kit (Life Technologies); libraries were processed in batches of eight samples per emulsion. Sequencing of the libraries was performed on Personal Genome Machine (PGM, Life Technologies) using the Ion 318 Chip and the Ion PGM Hi-Q Sequencing 200 Kit (Life Technologies).

### Data analysis and variant calling

Data analysis, including alignment to the hg19 human reference genome and variant calling, was done using the Torrent Suite Software v4.6 (Life Technologies). Filtered variants were annotated using a custom pipeline based on vcflib (https://github.com/ekg/vcflib), SnpSift [37], the Variant Effect Predictor (VEP) software [38] and NCBI RefSeq database. Alignments were visually verified with the Integrative Genomics Viewer (IGV) v2.3 [39].

### Analysis of callable loci

Analysis of callable/uncallable/poorly mapped loci was performed using the Torrent Variant Caller, to discriminate between variants which can be automatically detected from those that are hindered by sequencing errors due to homopolymers or PCR amplification artefacts. SNPs and small (<100 bp) INDELs spanning the coding regions of *BRCA1* and *BRCA2* were retrieved from the ClinVar and COSMIC databases in VCF format, converted to a Hotspots file and used to guide variant calling. In this way, the variant caller is forced to analyse a given hotspot coordinate; if there is no mutation, the software outputs that the position is “reference”; otherwise it outputs the mutation detected. If there are problems in the sequence at that position, the software outputs a “no call” value indicating if variant calling failed due to strand bias, low quality of bases, noise in the sequence, poor mapping. All the “no call” positions were further inspected by visual verification of the alignment file to ascertain whether the “no call' status was due to artefacts or homopolymer misalignment. This analysis was carried out for each of the 47 samples and the frequency of “no call” status for each hotspot coordinate was recorded.

### DNA Sanger sequencing

Mutations of *BRCA1* and *BRCA2* were validated by Sanger sequencing (primer sequences available upon request). PCR products were purified using Agencourt AMPure XP magnetic beads (Beckman Coulter) and labelled with BigDye® Terminator v3.1 (Applied Biosystems). Agencourt CleanSEQ magnetic beads (Beckman Coulter) were used for post-labeling DNA fragment purification, and sequence analysis was performed on the Applied Biosystems 3130xl Genetic Analyzer.

## Abbreviations

EMA: European Medicines Agency
FDA: Food and Drug Administration
FFPE: formalin-fixed paraffin embedded
FIGO: International Federation of Gynecology and Obstetrics
HR: homologous recombination
IGV: Integrative Genomics Viewer
NGS: Next-Generation Sequencing
PARP: poly(ADP-ribose) polymerase
PARPi: poly(ADP-ribose) polymerase-inhibitors
PCR: polymerase chain reaction
PGM: personal genome machine
SNP: single nucleotide polymorphism
